# A FEASIBILITY STUDY: THE DEVELOPMENT OF WEARABLE TECHNOLOGY FOR INJURY PREVENTION AMONG DIRECT CARE WORKERS

**DOI:** 10.1093/geroni/igz038.3062

**Published:** 2019-11-08

**Authors:** Yuchi Young, Yuchi Young, Mitch Leventhal, Jonathan Muckell, Peter E Raymond, Fred Erlich, Christopher Paynter

**Affiliations:** 1 SUNY at Albany, Albany, New York, United States; 2 SUNY at Albany, Engineering & Computer engineering, Albany, New York, United States; 3 The New Bureau, New York, New York, United States; 4 Living Resources, Inc., Albany, New York, United States

## Abstract

Objectives: 1) create metrics for lifting techniques and transferring mechanisms, 2) calibrate sensors for data collection 3) identify potential injurious posture among home health aides (HHAs) while transferring patients. Participants: 7 HHAs and a physical therapist. Interview and sensor data were collected. Outcome variables included improper lifting techniques and improper body mechanisms. Obesity of HHAs was associated with worse scores of body mechanics (p < 0.0001), while fear of injury with better body mechanics (p < 0.0001). GEE results identified that twisting the spine during transfers (OR = 6.3; 95% CI: 1.09–36.7) and not using a wide support base when lifting from supine to sitting (OR= 6.0, 95% CI: 2.03–17.7) were associated with improper lifting technique and body mechanics. Results show it is viable to use sensor technology to collect HHAs’ data to design intervention for injury prevention. A larger-scale study is needed to validate the results.

